# Continuity and Discontinuity in Artefact Distributions from Multi-Period Archaeological Sites

**DOI:** 10.1007/s10816-026-09821-0

**Published:** 2026-09-14

**Authors:** Eljas Oksanen, Andrew Bevan, Andrew Carter

**Affiliations:** 1 University of Reading, Reading, UK; 2 UCL, London, UK; 3 Independent Scholar, Norwich, UK; 4 University of Helsinki, Helsinki, Finland

**Keywords:** Spatial statistics, Archaeological citizen science, Landscape archaeology, Public finds, Diachronic change detection, England

## Abstract

Artefact finds recovered by metal detecting and reported by members of the public are a growing source of archaeological information, with over 1.2 million finds recorded to date by the Portable Antiquities Scheme in England and Wales. Heritage schemes in many other European countries where hobby detecting for archaeology is legal likewise report growing numbers of finds. Using point pattern analysis, this paper presents a worked-out set of computational methods to study diachronic processes within artefact assemblages at field or local (*e.g.* parish) levels. We present a simulated ‘toy’ example to illustrate the methodology and test it with two large artefact assemblages from the counties of North Yorkshire and Norfolk that differ in terms of finds record quality and known contextual archaeological landscape information. The proposed approach and example code can be reapplied to other archaeological point patterns. It formalises a spatial statistical workflow that produces an assessment regarding landscape persistence and reuse, and which can be used, for example, to assist fieldwork or planning decisions by identifying areas for further research. The case studies highlight the considerable need for formal analytical workflows in public finds research, which can be easily deployed at different sites and local assemblages. Moreover, while the proposed workflow has been designed with public finds scatters in mind, it is scale-agnostic and can be reapplied to investigate a range of archaeological phenomena from monument distributions across a larger region to investigating artefact scatters within an excavated area.

**Supplementary Information:**

The online version contains supplementary material available at https://doi.org/10.1007/s10816-026-09821-0.

## Introduction

This article considers what spatial statistical methods are most appropriate for examining spatial overlaps in multi-period archaeological sites and landscapes. Sometimes this kind of focus is termed an archaeology of landscape ‘persistence’ because it is interested not only in ordinary examples of continuity in activity across two successive time periods, but also in theoretical concepts of human place-making and in common but complicated examples of landscape reuse after a chronological hiatus (Schlanger, 1992: 92; Bevan & Conolly, 2013: 7–10, 217–221; Daubney, 2016: 39–45). We take the analysis of English metal-detecting finds as both a motivation and a source of two case studies, asking: How can the over hundreds of thousands of metal-detected objects reported by members of the public in England and Wales be used to locate and interrogate continuity and discontinuity in human activity at particular sites? Palimpsest surface or near-surface ‘scatters’ of finds from a ploughsoil zone are thus the primary vantage point in what follows, but it is also worth stressing that similar challenges often exist with excavated assemblages. In any case, the traditional statistical methods used in archaeology for assessing site location patterns, basic variations in findspot density or artefact type co-occurrence, are not especially helpful in cross-phase continuity in activity. We have therefore been motivated to consider a wider set of options, first via attention to a ‘toy’ simulation, and thereafter with a view to understanding two example sites comprising multi-period metal-detector finds. Specifically, we aim (a) to distinguish between multi-period scatters of random stray finds and of sub-scatters that are linked by more meaningful continuity of activity and deposition, as well as (b) to deploy statistical and computational methods to deduce diachronic patterns from the latter.

**Fig. 1:**
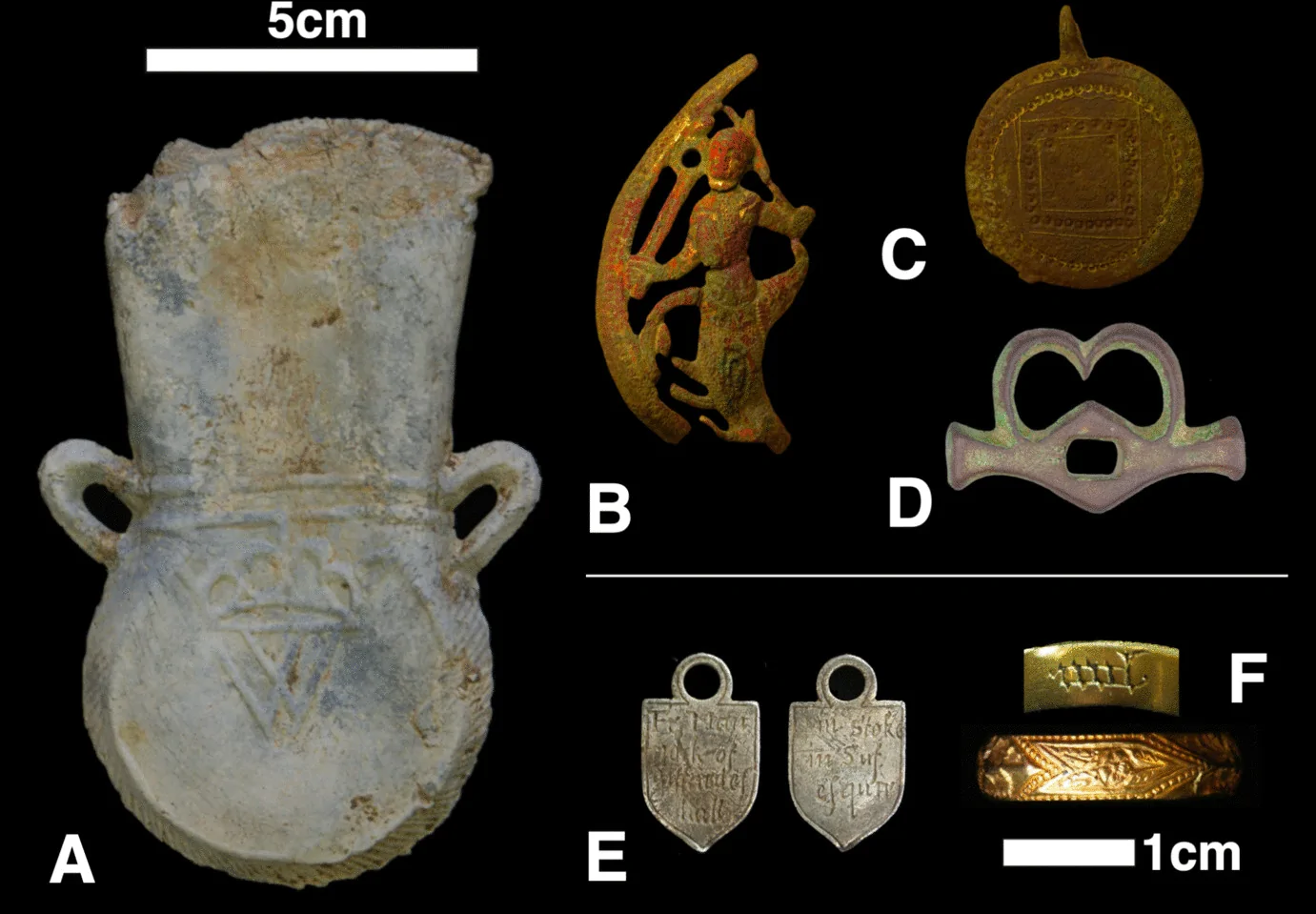
Medieval and post-medieval finds recovered and reported to the PAS by AC in the Foulsham parish. **A** Lead ampulla/pilgrim flask (PAS Unique ID NMS-D0E9F8). The crowned W suggests it came from the nearby Shrine of Our Lady of Walsingham in Norfolk, one of the most important pilgrimage sites in England. **B** High medieval enamelled and gilt copper-alloy Limoges mount depicting a mythological figure (NMS-910537). **C** Twelfth-century gilt horse harness pendant (NMS-8D1367). **D** Late medieval dagger guard (NMS-57D2E4). **E** A silver pendant shield, probably a vervel, naming Francis Mannock, a member of East Anglian gentry in the sixteenth or seventeenth centuries (grandfather or grandson of the same name, NMS-1E9786). (**F**) Late medieval gold ring with the inscription ‘nul auteur’/‘none other’ (NMS-EDEE62). Image credit: PAS. Images adapted from PAS, licence: CC BY-SA 4.0 Int

We draw our real archaeological datasets from finds registered by the Portable Antiquities Scheme in England and Wales (hereafter PAS), an archaeological heritage scheme for recording and managing public finds of all kinds, from the Palaeolithic to the modern day (though post-1700 CE objects are only selectively recorded).^1^ Records created by PAS Finds Liaison Officers (FLOs), detectorists with self-recorder status and other affiliated personnel offer a kind of first triage of new finds that later see further enhancement by particular finds specialists. They are particularly important for those artefacts uncovered by public metal detecting (Fig. 1 for examples recovered and reported by AC^2^). Metal detecting for archaeology is a regulated, but legal activity under English and Welsh heritage laws. Most PAS finds have been recovered on ploughed fields (92% of all finds in 2024 were reported from cultivated land, Lewis, 2025: 39) and therefore arrive from contexts that are not only archaeologically destroyed but within which objects are subject to ongoing degradation from corrosion and agricultural activity (Haldenby & Richards, 2010). Since 1997, and in particular since its full-scale implementation in 2003, the PAS has created over 1.2 million records consisting of almost 2 million individual objects (Bland, 2005; Lewis, 2016b; 2025; the numeric difference is made mostly by hoard finds). The two case studies that we develop in the latter half of the paper offer insight into (a) long-term settlement continuity from the Roman period to the medieval around the village of Well in North Yorkshire, and (b) land-use contraction following the fourteenth-century demographic collapse around the village of Foulsham in Norfolk, East Anglia. The two site assemblages are also differentiated by the precision of the recorded spatial findspot data, allowing us to compare the suitability of different techniques given different levels of data quality and published spatial resolution.

The goal of the case studies is to explore and test the robustness of certain reproducible statistical methods for investigating diachronic change at the level of local or parish assemblages (*e.g.* from the ground surface or from the ploughsoil). Almost 1 million individual findspots have been recorded at 6-figure British National Grid Reference or better (*i.e.* within a square 100 m to one side, or better; Lewis, M., et al., 2025) and therefore have spatial data of sufficient quality to be used for site-level studies. There have now been several surveys that explore the contents of the PAS database via more formal spatial analysis (*e.g.* Brindle, 2014; Gosden et al., 2021; Richards et al., 2009). Most of these have been, however, conducted and published using bespoke GIS software, with analytical workflows that are not easily reapplied and transferred to other sites. This paper therefore examines the challenges involved in producing site-level analyses using metal-detected material, emphasises a code-based method and valorises the still underexploited potential of the many PAS assemblages across England and Wales to shed light into the archaeological and landscape processes at a local level (*cf*. Brindle, 2014; Daubney, 2016; Robbins, 2012).

## Methods

Spatial point pattern analysis is a well-established field of statistics (*e.g.* amongst many treatments, see Baddeley et al., 2015, with further references), and there are well-known applications across a range of subjects, including archaeology (Bevan, 2020 for a summary). Here, we wish to introduce and clarify, with as little jargon or formal notation as possible, several spatial statistical concepts that are relevant to the research problem at hand, at the same time as introducing several specific point pattern methods that are potentially relevant.

### A Toy Workflow

In order to assess the effectiveness of different methods and to fix theoretical ideas a little more clearly, we start with a ‘toy’ simulation of an archaeological site involving finds from three hypothetical chronological periods. As noted above, we are motivated by the challenge of understanding a palimpsest surface or near-surface ‘scatter’ of finds coming from a ploughsoil zone. Although we focus on case studies from metal detecting below, we assume that these finds could in principle be lithics, potsherds, metal or other materials and that their locations have been slightly disturbed, but remain informative about localised patterns of past activity on the site. The simulation also provides full reproducibility in a way which is not possible for the case studies, because PAS protocols request publication only of reduced precision for findspot coordinates (to 4-figure National Grid References, *i.e.* to the nearest 1 km^2^ square).

Each period in the toy example shows evidence for several sub-areas of activity across the site. Some of these sub-areas show continuity from one phase to another, and some do not. There is also some degree of random noise in the spatial distributions that has been introduced to obscure the original patterning slightly. The simulation can be re-run within certain plausible parameters to create many different versions, but here, we focus on just one realisation, as an example.

**Fig. 2:**
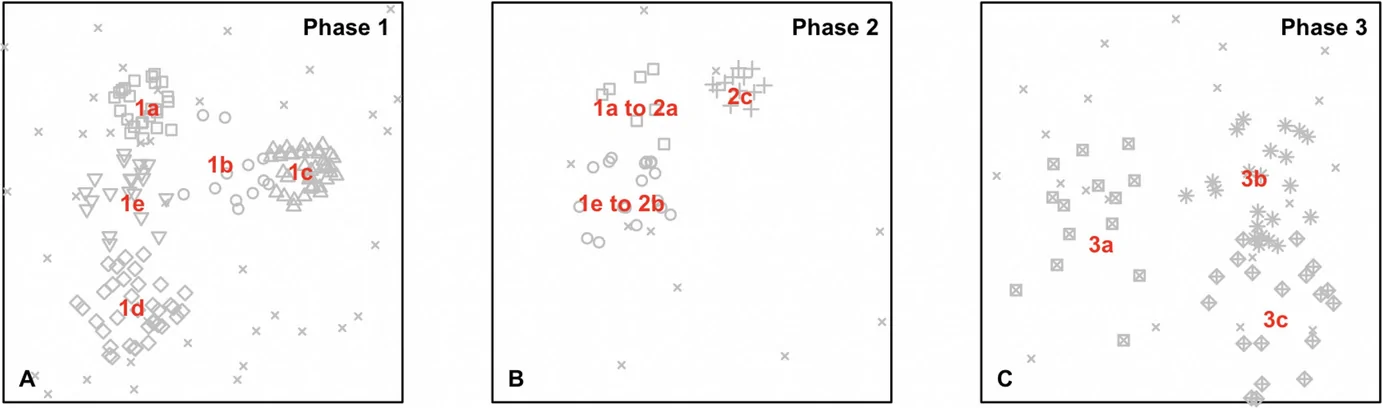
A ‘toy’ simulation of a surface artefact scatter across a hypothetical 250 × 250 m region, with three phases. The finds are shown with symbols indicating the scatter sub-area within which they were generated, and labelled in red (small crosses are added as ‘noise’)

Figure 2A shows the point distribution of a hypothetical phase 1 for this scatter and is made up of 5 sub-areas of activity. There is also some further noise added, for example, to imitate the impact of ploughsoil disturbance (Daubney, 2016: 84–88). Figure 2B shows phase 2 in which sub-areas 1a and 1e from phase 1 continue in use, while several others cease activity, with only one new sub-area at 2c. Figure 2C adds a third phase that assumes general site reuse without any continuity in particular sub-areas (3a–c are new).

### Kernel-Based Intensities

One common way to visualise a point pattern, especially a dense one where assessment by eye is tricky, is to calculate the first-order intensity in a series of localised moving windows (called kernels) that produce a final raster image that is often known in the GIS literature as a kernel density estimate (KDE, Conolly & Lake, 2006: 175–178). For this method, the kernel moves systematically across the study area, and points falling inside the kernel are counted up with the resulting total density then mapped at the temporary location of the kernel centre, before it is moved to a new location. The result, such as in Fig. 2A, is sometimes also colloquially called a ‘heatmap’. In many GIS packages, the choice of kernel is relatively simple, such as a circular area of fixed radius and hard edges, so that points falling outside that circle are not used in the local calculation. Sometimes there is a further option to down-weight points towards the edges of the circle (Bevan, 2020: 61–63 for discussion). However, more specialist software implementations (*e.g.* *density.ppp()* from the spatstat package in the R environment, Baddeley et al., 2015: 155–198) tend to favour continuous kernels that use every point in the dataset in every calculation, adjusted by some kind of inverse-distance weight. In the latter cases, a Gaussian kernel (*i.e.* a 2D bell-curve) is often preferred with the kernel size or bandwidth defined via a standard deviation.

**Fig. 3:**
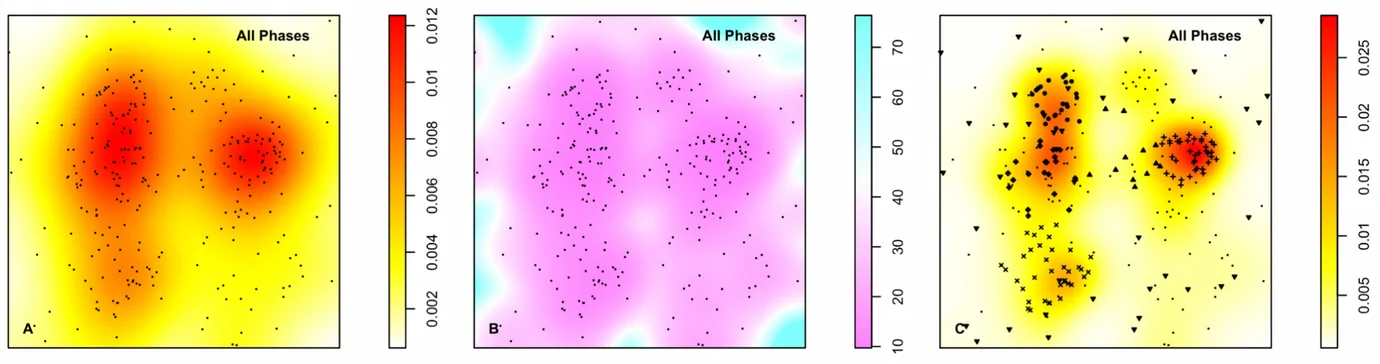
Examples of **A** a fixed kernel intensity estimate for the whole 3-phase toy dataset (Gaussian kernel, *σ* = 25 m bandwidth); **B** adaptive kernel sizes selected by an optimising routine (Gaussian kernel, variable ~ 10–75 m bandwidths); and **C** the adaptive kernel estimate for the whole 3-phase toy dataset (~ 10–75 m *σ*). The study area is hypothetically 250 × 250 m

Figure 3A shows a basic fixed-bandwidth, kernel-smoothed intensity surface (aka ‘kernel density map’) of the distribution. A recently introduced and useful alternative (Davies & Baddeley, 2018) is to fit kernels of varying bandwidths across the study area, smaller where the number of points is large and where there is more detail to discern, larger where the number of points is small. Figure 3B therefore shows an example of the different kernel sizes (standard deviations of a Gaussian kernel that vary from 10 to 75 m approximately) that are suggested by an automatic bandwidth selection routine for the simulated toy data. When these adaptive kernels are used, we get a result such as Fig. 3C, which offers greater insight on the size and shape of the high-density portions of the site scatter, while falling back to a smoother, coarser view of patterning where the data points are sparse.

### DBSCAN Classification

**Fig. 4:**
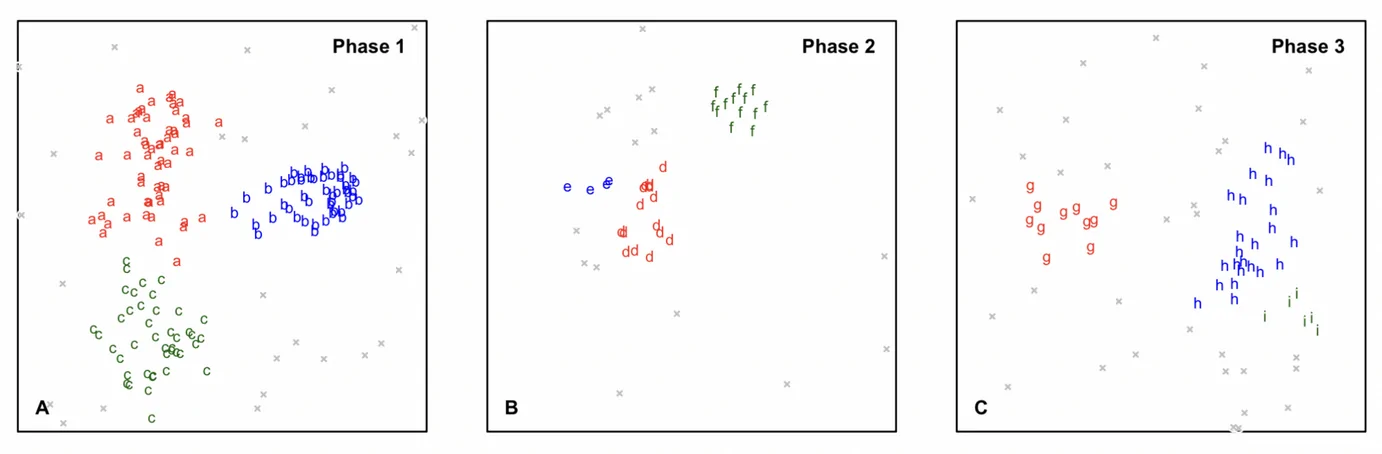
The same toy simulation of three phases, but the individual finds are labelled according to a dbscan clustering solution that reconstructs the original clusters from nothing but the spatial patterning for that phase (crosses are those reconstructed as isolated, ungrouped finds). The study area is hypothetically 250 × 250 m

One commonplace way to find and label spatial sub-clusters in a point pattern is via a density-based clustering algorithm such as DBSCAN (hereafter dbscan, Ester et al., 2009). Within archaeology, sub-cluster identification is often done informally, by eye, or simply with the visual aid of a kernel density map. However, there are at least a few formal statistical examples in archaeology that use dbscan or alternative methods (Bevan & Conolly, 2013: 225–226; Carrero-Pazos et al., 2019). Figures 4A–C show dbscan results for each of our three hypothetical phases in an attempt to reidentify the sub-clusters of this point pattern empirically. In our workflow, this can also be a helpful step for a user in terms of creating an initial visual assessment of broad cluster structures in a complex point pattern, highlighting aspects that simply ‘eye-balling’ the distribution does not easily reveal. While the results reassuringly suggest polyfocal activity in each period and do roughly recover many of the pre-cooked activity sub-areas in each phase (compare with Figs. 2A–C), they provide inevitably a fairly blunt approach. Notice also that we introduce this method here and use some arbitrarily chosen parameter thresholds (see Birant & Kut, 2007 for further discussion) without carrying it forward to the real analysis, because of the complications introduced by the rounding of spatial coordinates in the PAS data which we feel limits its use in this particular case. However, the technique remains relevant in other situations.

### Relative Risk

The general idea of combining two kernel-smoothed estimates of first-order point intensity in order to map spatial ‘relative risk’ was first developed for epidemiological applications (Bithell, 1991; see also Kelsall & Diggle, 1995) and has more recently also been applied to archaeological evidence (Bevan, 2012: 500–504; Monna et al., 2013: 512–514; Smith et al., 2015: 367–369; Nenova, 2019: 89–98, see also Bevan, 2020: 67–69 for discussion). The basic idea is to respond to the unevenness of real-world distributions by contextualising a subset of population (*e.g.* Roman-era finds) against another subset or the entire sample. In our example, detectorists may have searched intensively one area but only lightly an adjoining one. A seemingly high number of Roman finds in the former may simply be the result of this enhanced local recovery rate, but the same number of finds in the latter is more likely to signify—in relative terms—more intensive depositional activity during the Roman period.

**Fig. 5:**
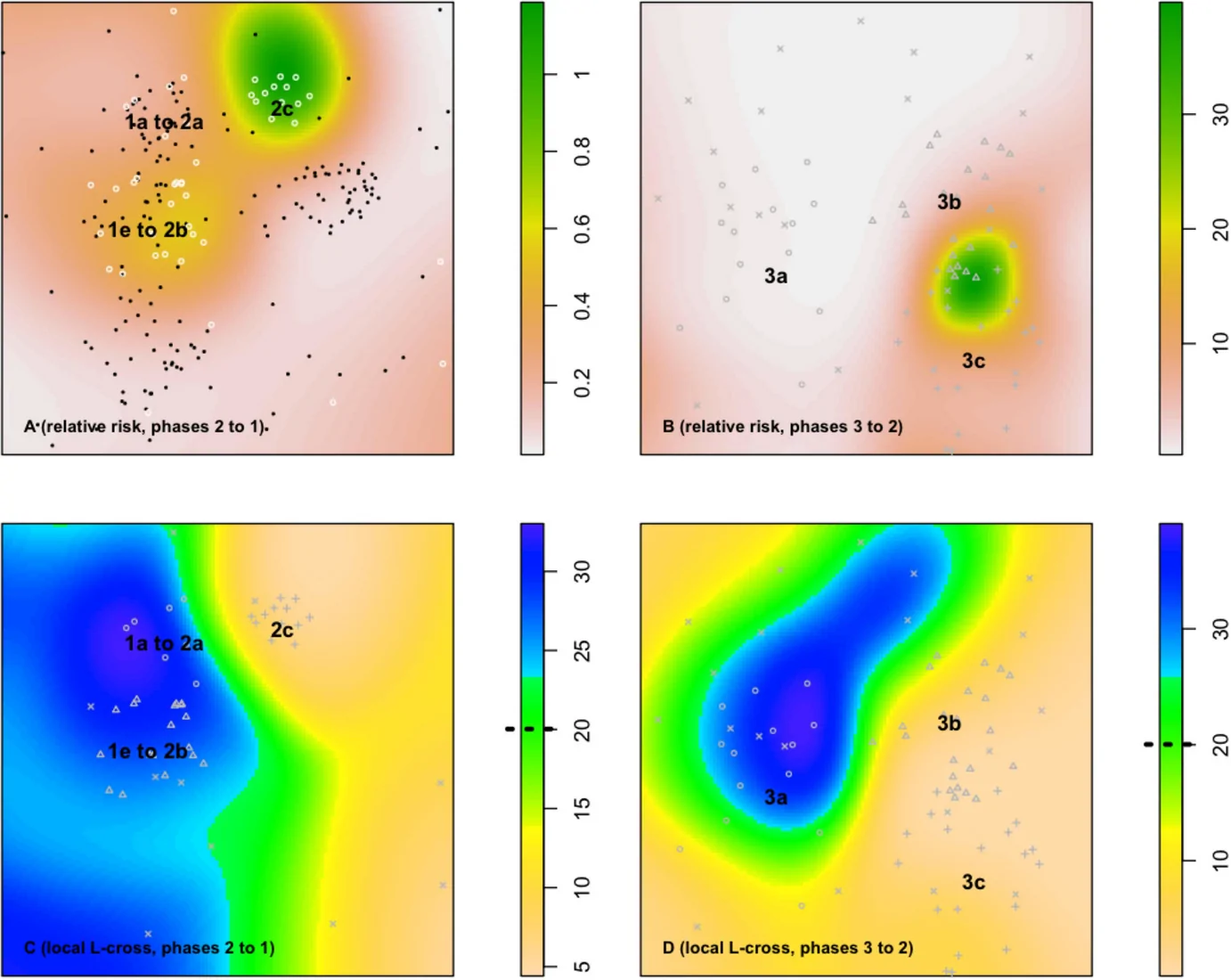
Two ways to visualise continuity or discontinuity: **A**, **B** a ‘relative risk’ surface (1 is the threshold value between over- and under-representation) and **C**, **D** an interpolated L-cross function at radius 20 m (therefore, theoretical CSR is 20, at the dotted line). The study area is hypothetically 250 × 250 m

Figure 5A provides an example where each pixel in the kernel density surface of phase 2 in the toy example has been divided by the value in the corresponding pixel from the phase 1 kernel density surface, in order to produce a map of the ratio of phase 2 finds to phase 1 finds across different portions of the study area. While this example figure shows the resulting surface for the entire study area, and we recommend that this surface is always examined, there remains a good case for cropping out those areas that are subject to spurious statistical anomalies because the denominator of the ratio is close to 0. In the case of real metal-detecting data, this can be useful for controlling significant outliers, such as when a detectorist examines intensively only one small, isolated area in a region that produces finds of only one period. ‘Adaptive’ kernels (as above) and ‘shrinkage’ estimators (Bithell, 1991) provide alternative ways to handle such sparse portions for the map for relative risk estimates, but we have chosen to keep things simple in what follows. In the context of our study, a relative risk surface therefore measures the local ratio of finds belonging to one period compared to finds in the same local neighbourhood of a single other period. More concretely, if we marked out a circle in a ploughed field with a radius of 20 m and then metal-detected that area, what would be the ratio of finds from phase 2 to phase 1? In Fig. 5A, a higher value (marked by darker pixels on the colour scale) indicates a higher proportion of phase 2 finds in the local area. On the colour scales, the value of 1 means the target subpopulation is represented at the same rate as the reference population (*e.g.* period 2 vs period 1 finds), with higher and lower values an approximate multiplier for over- and under-representation. In our example, the method picks up on the depositional continuity between 1e and 2b, but this is less well evidenced between 1 and 2a, as proportionally fewer finds belong to 2a.

### Local L-Cross Function

K functions represent a technique that assumes the first-order intensity we have been discussing above is relatively homogeneous across the study area, and instead that we wish to understand how interactions among the observed points might lead to second-order, clustered or dispersed patterning at different scales (or conversely might just exhibit complete spatial randomness, CSR). Clustering could theoretically indicate people returning to an area possibly for similar reasons as their antecessors, and dispersal that behavioural activity patterns had changed across time.

Basic K functions and their many relatives (L functions, pair correlation functions, G functions, J functions: see Baddeley et al., 2015: 119–296) have already seen considerable use in archaeology (*e.g.* initially, Bevan et al., 2004; Orton, 2004). Here, however, we pay attention to an as yet unused kind of K function analysis that is helpful in situations (a) where the above assumption of study area homogeneity is violated, (b) where one is interested in localised, mappable patterns of clustering or dispersal rather than a single global pattern, and (c) where the dataset is multi-type, for example involving comparison of how Phase 2 finds might be clustered or dispersed relative to Phase 1 finds. Nakoinz and Knitter (2016: 140–142) have already briefly advocated a bespoke use of a G function in a fixed window for a similar goal. Here we merely agree with their rationale, but prefer a version based on a ‘local K-cross function’ that already has good practical and theoretical support (Getis & Franklin, 1987; Baddeley et al., 2015: 595). K-cross function is the best supported in the literature, and for this study, we simply use a linearized version of this (L-function) for ease of visual interpretation.

Figure 5C and 5D shows an example implementation of a local L-cross function with a local 20 m radius neighbourhood where we have interpolated the result, so it is more easily compared to the kernel density and relative risk surfaces. The benchmark value for independence between observations (theoretical complete spatial randomness) is the same as the value for the radius at which the function will be computed at each point (so here 20 m). Below this value, the point process trends towards dispersal/regularity, and above towards clustering. This visualisation successfully models the two sub-scatters in the toy simulation that have been engineered to exhibit continuity between phase 1 and 2 (Fig. 5C, 1a to 2a, 1e to 2b), and it reassuringly does not emphasise those sub-areas that are either new or have been abandoned. In other words, the left half of the image emphasises clustering, and the right dispersal. This is in many ways a more helpful perspective than the one offered by the relative risk surface in Fig. 5A, where it is the new sub-scatter from phase 2 that is visually emphasised (2c). That said, Fig. 5D sounds a warning that there is nothing magical about such a technique, as it merely indicates overlaps between two types of points. Hence, it is also subject to random chance overlaps, such as the new sub-scatter 3a which just happens to be located in the midst of phase 2 finds, but was not designed to be so. We nevertheless argue these techniques identify and summarise patterns that are not readily apparent by visual inspection only. Moreover, deploying different methods operating on different statistical principles (relative risk surface and bivariate local L-cross function) allows for a more complete interpretation of an assemblage.

## Portable Antiquities Scheme Assemblages

The above hypothetical example provides a good starting point for thinking through some of the inferential problems involved, and so we now turn to two real case studies. There is a tremendous amount of potential for computational and diachronic research using PAS evidence, and as already noted, it has been investigated both by major research projects (*e.g.* Gosden et al., 2021; Richards, Naylor & Holas-Clark, 2009) and several individual studies at national, regional and local scales (*e.g.* Bevan, 2012; Kelleher, 2013; Cool & Baxter, 2016; Daubney, 2016; Oksanen & Lewis, M, 2020; Webley, 2020). We will next discuss what conclusions can be drawn from two case studies that examine diachronic change and continuity at two sites. We will then consider the implications for exploiting the available public finds data.

**Fig. 6:**
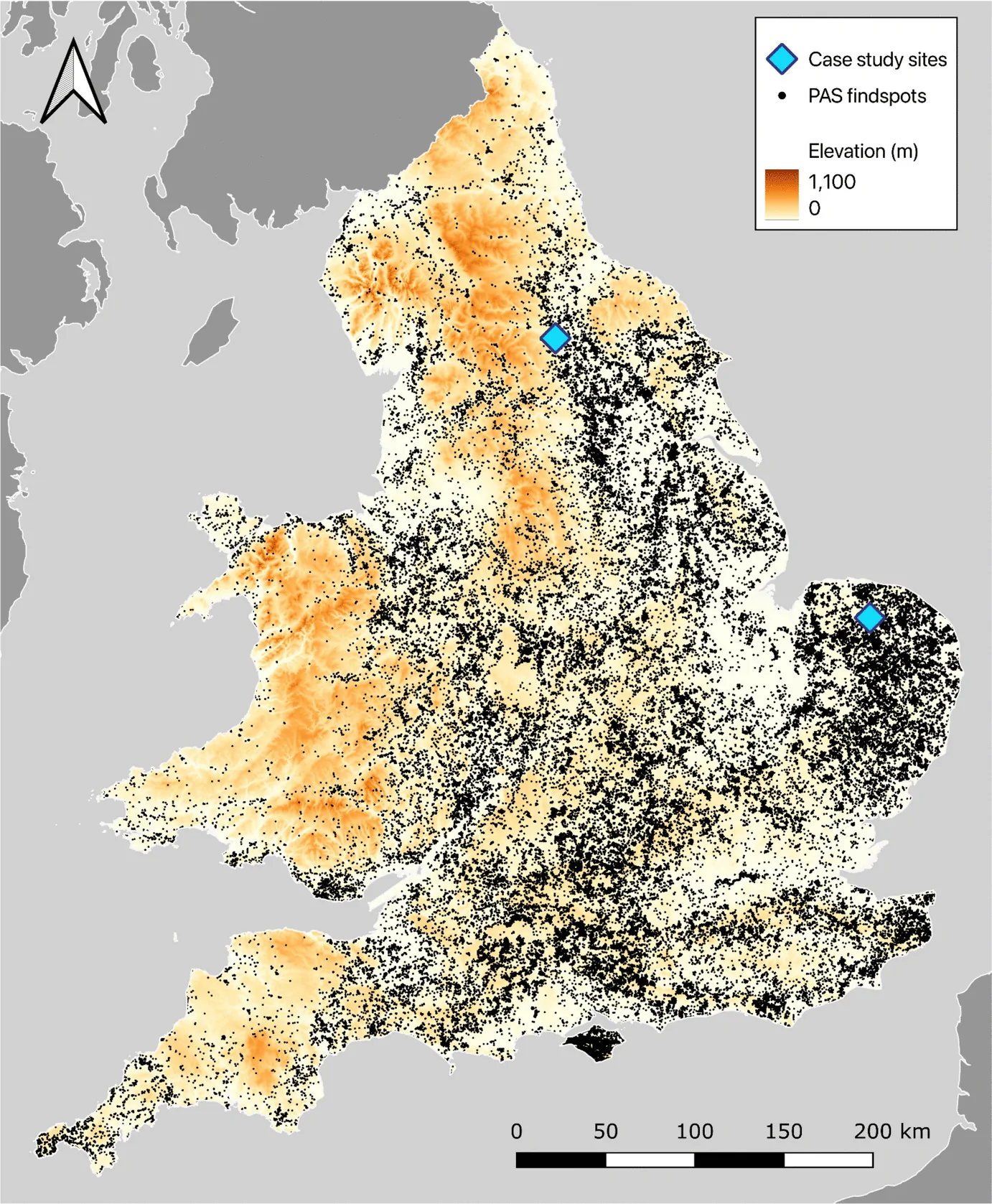
The distribution of PAS findspots with known coordinates (*n* = 954,786) recorded up to June 2023, overlaid on an elevation model for England and Wales, with the two case study sites of Well, North Yorkshire, and Foulsham, Norfolk. Data: PAS, Mapzen Global Terrain

The two case study sites that follow in this paper come from Foulsham in Norfolk and Well in North Yorkshire (Fig. 6). They have been chosen deliberately to represent different kinds of metal-detected assemblages. They exhibit different finds of varying chronological date, with findspots recorded at differing levels of spatial precision (Tables 1 and 2), and they are manifest at slightly different scales (*e.g.* parish versus a few fields). Furthermore, over 90% of the finds at Foulsham were reported by a single detectorist (AC, and co-author here), while almost all of the finds at Well were produced by many people participating in several detecting rallies (typically 1–3 finds each). As will be discussed, the amount of other archaeology recorded at each site likewise varies.

**Table 1:** Chronological breakdown of finds recorded at the two case study sites by FISH thesaurus main chronological periods. Data: PAS

*FISH / PAS broad period*	*Foulsham*	*Well*
Other prehistoric	20	4
Iron Age	12	0
Roman	315	39
Early Medieval	102	10
Medieval	636	78
Post-medieval	390	111
Other	7	5

**Table 2:** Breakdown of finds at the two case study sites by recorded spatial precision. A set of 10-figure British National Grid Reference (NGR) coordinates locates a findspot into a square 1m to a side, 8-figure to 10m square, 6-figure to 100m square, 4-figure to 1km square, and 2-figure to 10km square. Data: PAS

*NGR figure*	*Foulsham*	*Well*
10	1344	142
8	89	12
6	0	94
4 or 2	49	7

We did not select either of the case study sites because of any preconceived expectations of a particular result. They were all identified by exploring all PAS finds from the counties of Norfolk and North Yorkshire with a view to discovering significant local assemblages using a method for identifying anomalous concentrations in relation to the regional background scatter as developed by Cooper and Green (2017). These counties were targeted for study as Norfolk has the largest number of recorded PAS finds, while North Yorkshire covers a large region of varied archaeological landscapes, the relationship of which to public finds is not as well studied as in many other parts of England (see also Gilchrist et al., forthcoming, for the wider research context in which this project took place). To protect exact recovery locations, the results are presented as analytical map surfaces without exact findspots (although full access to these findspot data is available from the PAS, subject to a formal research project request).

### Well, North Yorkshire

The first case study involves a rich multi-period assemblage near the village of Well. The wider landscape around the village is richly layered and combines historic buildings, further features such as topographic irregularities on the land surface, and metal-detected finds. Well is less than 3 km north of the important Neolithic and Bronze Age complex at Thornborough Henges, a sequence of three aligned earthwork henges sometimes called the ‘Stonehenge of the North’. Well was also an important local centre in the Roman period, whose imprint is said to have continued to influence the shape of medieval tenurial organisation across nearby townships (Moorhouse, 2004: 28–29). The Well case study is therefore a useful test of whether Stephen Moorhouse’s assertion of long-term continuity in the wider area is supported by PAS evidence at a field-level site.

**Fig. 7:**
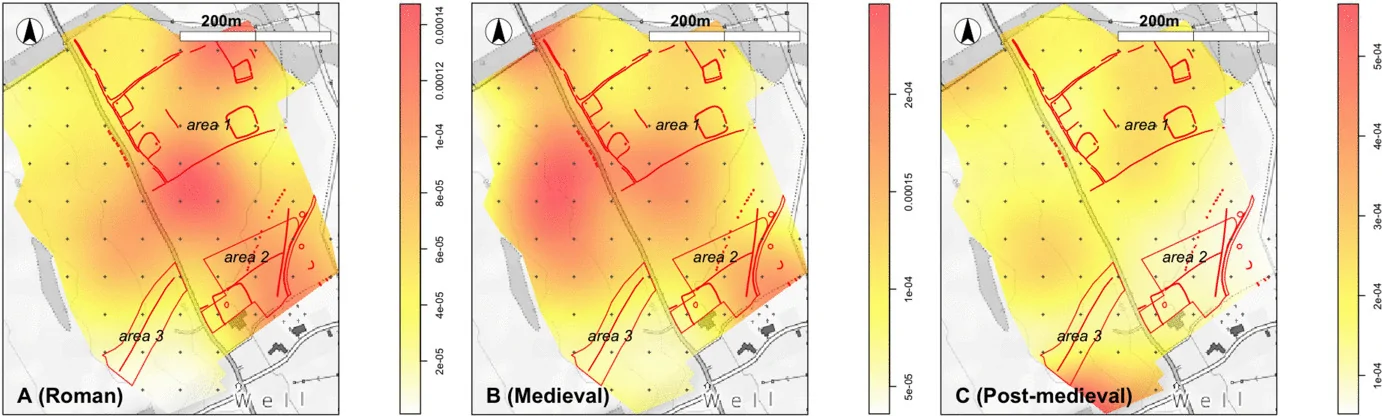
Well case study with cropmarks. Adaptive kernel density of **A** Roman finds (*n* = 39); **B** medieval finds (*n* = 78); **C** post-medieval finds (*n* = 111), with initial distance argument set at *h*0 = 100 with variable ~ 68–500 m Gaussian kernel bandwidths. National Grid Reference system grid corners at 6-figure NGR (100 m squares) have been added as small crosses. Data: PAS, Aerial Archaeology Mapping Explorer/Ordnance Survey. Basemap by OpenTopoMap

We focus on a field area just north of the village (Fig. 7) that has a substantial multi-period scatter of 248 metal-detecting finds recorded at minimum 6-figure NGR spatial precision. The field additionally contains several features identified through cropmarks in aerial photographs ranging from the Iron Age to the Modern period, reflecting the particularly deep-time character of the landscape. These include in the north-eastern quarter of the field (marked as area 1) Iron Age and Roman enclosures, trackways and field boundaries; in the south-eastern quarter (area 2) overlapping prehistoric, Roman or undated trackways, barrows, ditches and macula (round, unidentified cropmark), a Roman enclosure and post-medieval ridge and furrow; and in the south-west (area 3) post-medieval ridge and furrow.

We compare Roman (defined by PAS as 43–410 CE) with medieval (1066–1540 CE) and post-medieval (1540–onwards) finds to study long-term continuities in land use across the area. A preliminary assessment of the cropmarks appears to indicate that the division of the field area into broadly eastern and western halves by the modern road may reflect long-term continuity. This is seen in the general layout of the Iron Age/Roman enclosures and trackways, as well as the edges of the post-medieval ridge and furrow that abut the road. As a first step, the finds on the field were divided into east and west groups along the road, and a statistically significant difference was observed in the distribution of Roman (27 west, 12 east) versus medieval to post-medieval finds (98 west, 91 east) (*X*^2^, *p* = 0.04708; Agresti, 2013: 75–86). This suggests a general hypothesis that is consistent with the cropmark evidence, indicating that different depositional processes may have taken place in specific parts of the area in respective periods, specifically with an even pattern of later finds, but a less even pattern of Roman finds.

We next apply the point pattern workflow to the finds data to further explore the hypothesis. Because a large portion of the finds are recorded at only 6-figure NGR, we set the initial spatial distance argument in the function *bw.abram()* at *h*0 = 100 (see R code), resulting in a coarser adaptive kernel that varies between SD = 68 m in denser areas and SD = 500 m in the least dense. While somewhat arbitrary, we find this to be a good minimum range to examine the PAS finds distributions at Well given the variety in spatial findspot precision (Table 1). The precision of the spatial data (points stacked at a single coordinate location) must impact on the resulting map surface, but this does not appear to dominate the results with density hotspots loosely focusing between rather at the 100 m grid corners. In both case studies, findspots are also jittered very slightly (up to 2 m) to help break up the stacks; such artefact movement could be expected in the ploughzone. For the local L-cross function, the distance value for which the function should be computed (*i.e.* the ‘search radius’) is set at 200 m. The minimum radius that can here be applied to 6-figure NGR precision data is the diagonal of a 100 × 100 m square (141.4 m, so that the radius covers the entire square), which we round up to the nearest hundred. From the kernel density map (Fig. 7), we can see that the Roman findspots loosely concentrate in the eastern half of the field area, and the later finds in the western. This fits the Chi-squared test results, but is interpretatively useful only at a general level.

**Fig. 8:**
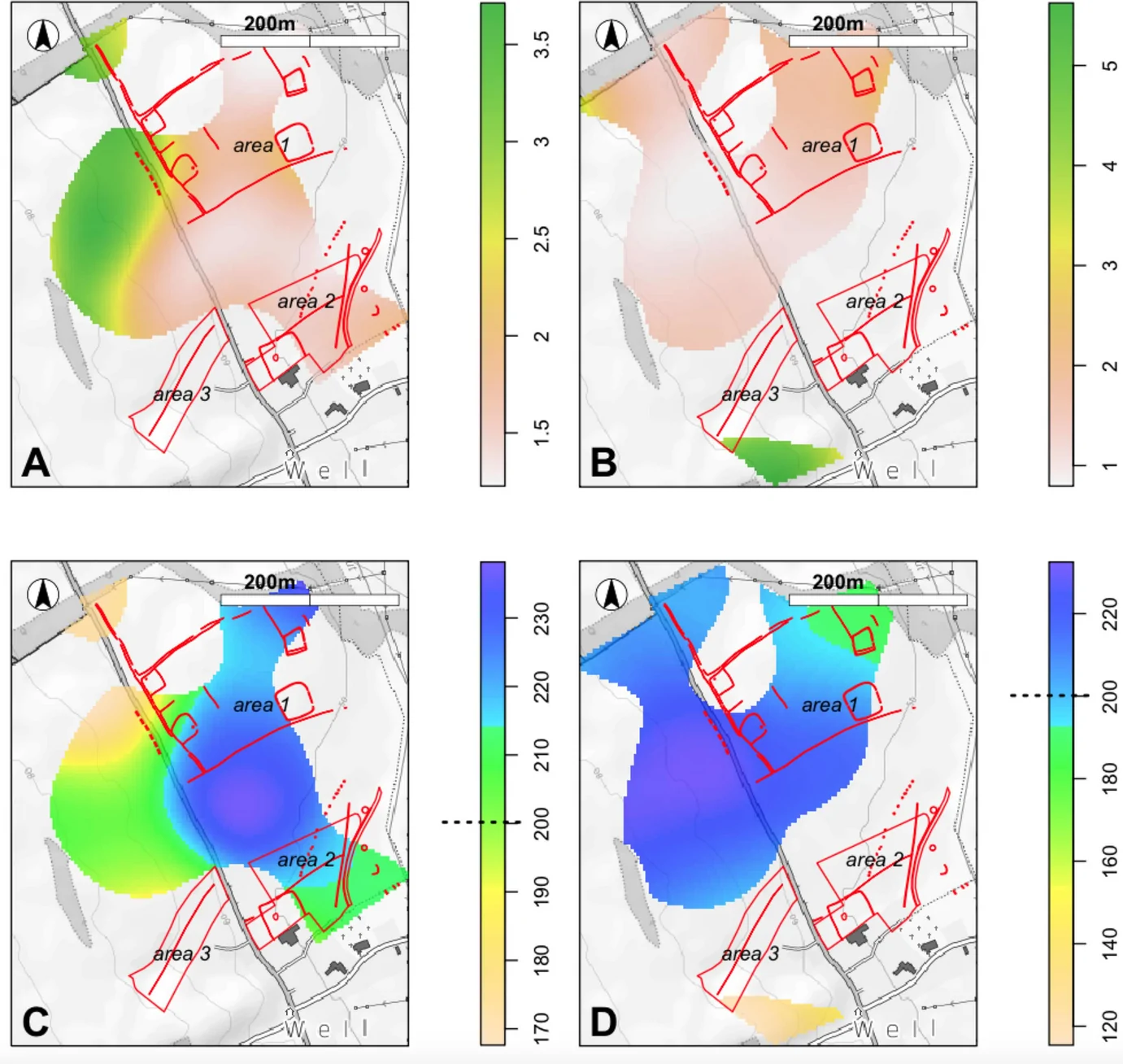
Well case study with cropmarks. Relative risk surface of **A** medieval to Roman finds and **B** post-medieval to medieval, based on adaptive kernel densities. Local L-cross function of **C** medieval to Roman finds; **D** post-medieval to medieval with *r* = 200 m, setting the theoretical value for CRS to 200, at the dotted line. Data: PAS, Aerial Archaeology Mapping Explorer/Ordnance Survey. Basemap by OpenTopoMap

Comparing the relative intensity of the medieval finds against the Roman, the former indeed focus on the western side of the study area. As noted above, we clip the surface to the highest-density findspot areas. Post-medieval finds are proportionally most prevalent near the settlement, and particularly so near the ridge and furrow cropmark in area 3 (Fig. 8). This fits with our hypothesis that the main foci of depositional activity shifted across different periods. From a long-term continuity perspective, however, the local L-cross approach indicates that—despite the overall focus of the later finds in the western half of the field—specifically diachronic clustering is most prevalent across the eastern through the central (medieval to Roman) and in the western through the central (medieval to post-medieval) parts of the field area. With particular reference to the earlier phases area 2 is where the time-depth of overlapping cropmark series is also at its deepest (prehistoric to post-medieval). We therefore interpret these results as reflecting Moorhouse’s argument of long-term continuity in landscape use around Well, at least if considered at large scale.

In conclusion, the analysis shows how the spatial focus of depositions at the study site shifted from one period to another across the Roman (probably extending from the late Iron Age) to post-medieval periods, but some areas retained an important (from a statistical perspective) pattern of reuse. This highlights the utility of applying different computational techniques to the data, with further validation from independent archaeological evidence (here cropmarks) to which the finds distribution appears to be connected.

### Foulsham, Norfolk

The second case study investigates shifts in activity patterns around the modern village of Foulsham. The parish has an unusually large assemblage of high spatial precision finds at 8-figure NGR or better (Table 1). Furthermore, we are not aware of any extensive archaeological landscape information being available from the Foulsham area in terms of published professional fieldwork reports, or areas of cropmarks identified from aerial photographs that are openly available. The PAS data therefore represents a very significant opportunity to better understand long-term processes related to the historic settlement. The hypothesis tested in this use case is whether settlement-level land-use patterns changed between major chronological periods, and in particular whether they contracted following the arrival of the Black Death plague pandemic (1348 CE in England) and transition from the high to the later medieval period around the fourteenth century. The analytical focus is therefore on applying a spatial point pattern workflow to understand parish-level processes in landscape use and exploitation, with specific attention to high-to-late medieval transitions.

Most of the PAS finds from the parish are the work of one of the authors, who has detected in the area with landowner permission since 1999 and has reported and/or self-recorded 1335 of the finds in our research dataset. Due to the European Union’s set-aside policy in the 1990s and 2000s (which removed a percentage of arable land from production), it was possible to detect particularly intensively and thoroughly on the fields visited. While detecting conditions always vary (*e.g.* soil moisture impacts on metal signals), the distribution of findspots should give a fairly reliable sense of variation in finds density across the area. It would appear that the majority of portable metal objects in the ploughzone have now been recovered and recorded, as more recent visits have yielded few finds. Given the parish-level spatial scale of this study and map visualisation, we have elected to use a similarly sized adaptive kernel as in the Well case study. However, the NGR spatial recording precision would make future examinations of finds densities at specific field sites possible to a much greater level of detail. For this reason, we have also tested using a distance value of only 20 m for the local L-cross function, resulting in a greater degree of granularity in examining localised finds patterns

**Fig. 9:**
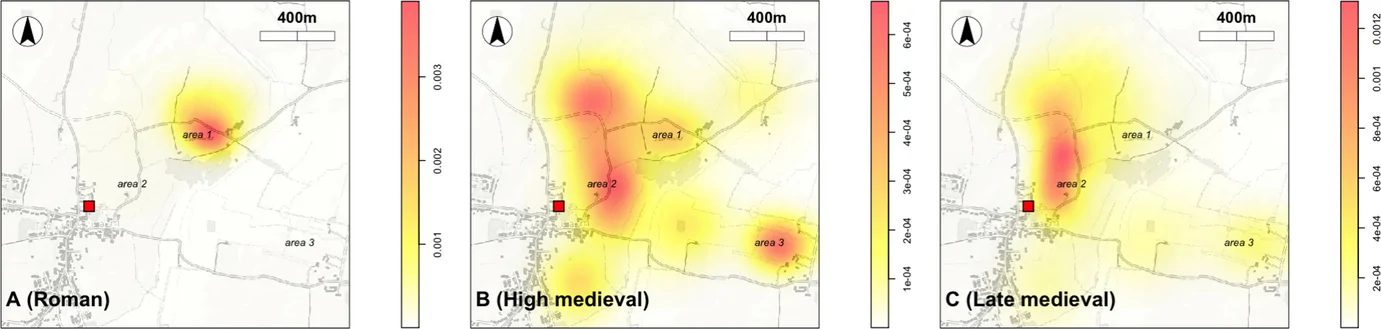
Foulsham case study area. Adaptive kernel density of **A** Roman finds (*n* = 310); **B** high medieval (*c*. 1100–*c*. 1300 CE, *n* = 288); **C** late medieval (*c*. 1400–*c*. 1600 CE, *n* = 315) finds, initial distance argument set at *h*0 = 100, same as at Well. For ease of reference, the location of the settlement’s medieval church of Holy Innocents is marked with a red square. Data: PAS. Basemap by OpenTopoMap

Figure 9 applies an adaptive kernel density estimation to the Foulsham PAS finds assemblage to highlight a clear shift in the intensity of depositional focus from the Roman (phase 1 at *n* = 310 with finds at 8-figure NGR or better) to the high medieval (dated *c*. 1100–*c*. 1300 CE, phase 2 with *n* = 288 finds) and later medieval (dated *c*. 1400–*c.* 1600 CE, phase 3 with *n* = 315 finds) periods. A temporal gap was left between high and late medieval period finds to minimise any overlap caused by uncertainly dated objects. Roman finds heavily concentrate on field areas to the north-east (area 1) of the medieval and modern settlement marked by the medieval church. In addition to metal objects, floor and ceiling tiles as well as Romano-British ‘grey ware’ sherds were recovered in this area, suggesting the site was a farmstead (Carter, 2020). It appears to have been the sole significant focus of Roman activity on the detected fields. Overall distribution shifts from the high to the later medieval period are more difficult to assess from the adaptive kernel density results. These finds congregate especially to one side of the village (area 2), extending out further east *c*. 1.3 km from the location of the medieval church. Here, a relatively high-density region of high medieval finds in the south-eastern quarter of the study area (area 3) declines in the Later Middle Ages. The areas of most intense distributions through the Middle Ages are near the settlement, and in many other areas, the high and late medieval object findspots are mutually intermingled much more so than with the Roman.

**Fig. 10:**
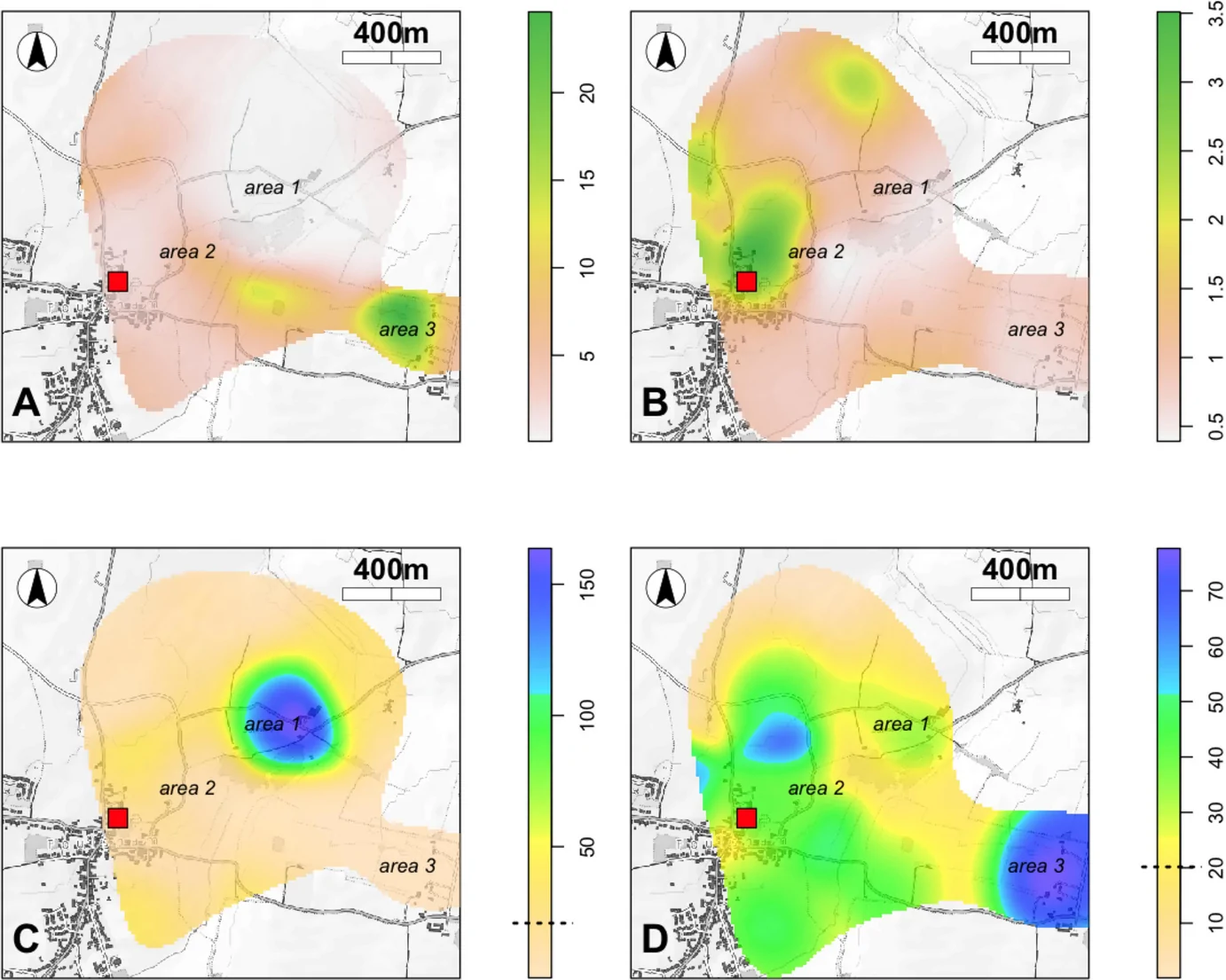
Foulsham case study area. Relative risk surface of **A** high medieval to Roman; **B** late to high medieval finds, based on adaptive kernel densities. Local L-cross of **C** high medieval to Roman; **D** late to high medieval finds with *r* = 20 m, setting the theoretical value for CRS to 20 at the dotted line. The location of the medieval church of Holy Innocents is marked with a red square. Data: PAS. Basemap by OpenTopoMap

The relative risk surface (Fig. 10) confirms what can already be assessed from Fig. 9, but also highlights the fact that the south-eastern cluster (area 3) has particularly few Roman finds. The relative risk surface of phase 2 and 3 finds shows that later medieval finds tend to be recovered closer to the settlement (area 2). This could indicate a contraction in the field system, or perhaps some other transformation such as outlying fields being turned into pasture.

The local L-cross results support shifts in the focus on depositional activity. First, Roman and medieval finds cluster most intensely only at the site of the proposed farmstead (area 1). This indicates there was a fundamental reorganisation of the human landscape between the Roman and medieval periods. Second, most areas of phase 2 and 3 clustering are close to the settlement (area 2). The PAS findspot data appears to be in line with a process of spatial contraction (to areas nearer the settlement core) in landscape exploitation that occurred around many settlements during the Later Middle Ages; in eastern England, this has been quantified especially by Carenza Lewis’ work on ceramic evidence from hundreds of individual sites (Lewis, C., 2016a: 795; 2020: 41–43). Our analysis similarly supports the view that different parts of the parish may have experienced different archaeological and landscape processes as one moves from the pre- to the post-pandemic period.

The south-eastern finds group at Foulsham (area 3), set well away from the settlement core marked by the medieval church, also shows relatively high spatial clustering between the high and late medieval periods. This was not strongly indicated by either the basic kernel density map or the relative risk surface (which are based on numbers of finds). Faden’s Map of Norfolk dated to the 1790 s shows that this cluster abutted a large common, located between Foulsham and the smaller settlement of Themelthorpe 2.5 km to the east. It probably marks a small satellite settlement or hamlet of cottages that grew by the medieval common in order to enjoy better access to it (Donald & Faden, 1797; *cf*. also Macnair, 2003 for an assessment of post-medieval land use at Foulsham). Judging by both the absolute and the relative (to the total period samples) number of finds, this settlement declined from the high medieval (*n* = 76 finds) to the late medieval (*n* = 18 finds) phase. The local L-cross results, however, suggest some level of continuity in site use as the earlier and later finds are strongly spatially clustered within their limited area. In other words, cross-period depositional activity continued, if at a lower level of intensity.

In conclusion, it is now possible to start teasing out details from the metal-detected findspot pattern, yielding useful information on landscape use across broad periods. The results pick out other potentially interesting areas of either intensive diachronic clustering or dispersal. They show how the focus of depositional activity clearly shifted from the Roman to the high medieval period, and how in the Later Middle Ages the deposition pattern follows a regionally well-known process of spatial contraction towards the settlement core—although with interesting if diminished continuity particularly in area 3.

This analysis is agnostic on how the finds may have ended up in these fields, whether being accidentally dropped by people working on them, deliberately deposited as part of some activity that took place there, or as the result of manuring. For verification, fieldwork, re-examination of any available remote sensing data and further study of the recorded assemblage would be necessary. However, the metal-detected finds data and these initial analyses have already suggested hypotheses for testing and specific areas for deeper examination.

## Discussion

The two case studies discussed above have allowed us to explore important methodological and theoretical issues associated with persistence, continuity and discontinuity in landscape use. To summarise, the goal has been to create an open, code-based spatial statistical approach to these issues at site-scale in order to:

1. Create rapid spatial and diachronic assessments of potentially large finds scatters.
2. Identify relevant patterns from the mass of the data, for contextualising against archaeological or historical information.
3. Select potential areas of interest for further study.

One important characteristic of metal-detected find records is their variable spatial precision. As already noted, the finds at Well are mostly from detectorist rallies in which coordinates are recorded with as coarse as 100 m (or 6-figure NGR) spatial precision (see Table 1). The PAS issued formal guidance to record findspots to 8-figure NGR only in 2017 (PAS, 2017: 3). Of course, many public finds assemblages, especially if the records are old, are not so precisely reported. Our results reveal the limitations of the analysis when the spatial precision of the data is poor. A third of the finds from the site at Well are reported at 6-figure NGR, which also means that any findspots from the same grid square are gathered into a single coordinate point ‘stack’ in the lower left-hand corner of a 10,000 m^2^ square (see Fig. 7), which inevitably has at least some impact on the density calculation. The actual density of the finds is hidden from someone carrying a simple visual inspection of coordinate points on a map, and this artificial stacking can furthermore skew subsequent spatial statistical analyses. It is because of this that we coarsen the spatial parameters used in the Well case study, but that is by no means a perfect fix. Despite these caveats, we would nevertheless argue that PAS spatial precision, even at 6-figure NGR, is often sufficient to reveal broad patterns of depositional activity and is still useful in examining other archaeological features—in this case cropmarks.

A related limitation to our work has been the PAS restriction not to publish the precise findspot coordinates. This study has therefore been an exploration of how to deploy such material in spatial statistical studies: our solution has been to focus on constructing map surfaces for purposes of illustrating and interpreting trends and archaeologically meaningful relationships. While we cannot make our case studies reproducible (*cf*. Marwick, 2017) without the data, the aim has nevertheless been to promote this principle by advancing open, repeatable code-based research practices in archaeology. Finally, although it is not necessary for examining broad trends among the findspot patterns studied in this paper, it is possible to simulate the spatial imprecision by randomising the findspot locations by a value equivalent to the stated NGR precision and then conducting a sensitivity analysis of the result. This could be useful if, for example, too many points were located at low NGR resolution grid corners. That said, we strongly advocate reporting and recording findspots to the highest precision possible. It is critically important, moreover, that machine-readable metadata (*e.g.* attribute values) on varying levels of spatial precision are included in finds records, so that the appropriate methods and parameters can be selected in a spatial statistical study.

**Fig. 11:**
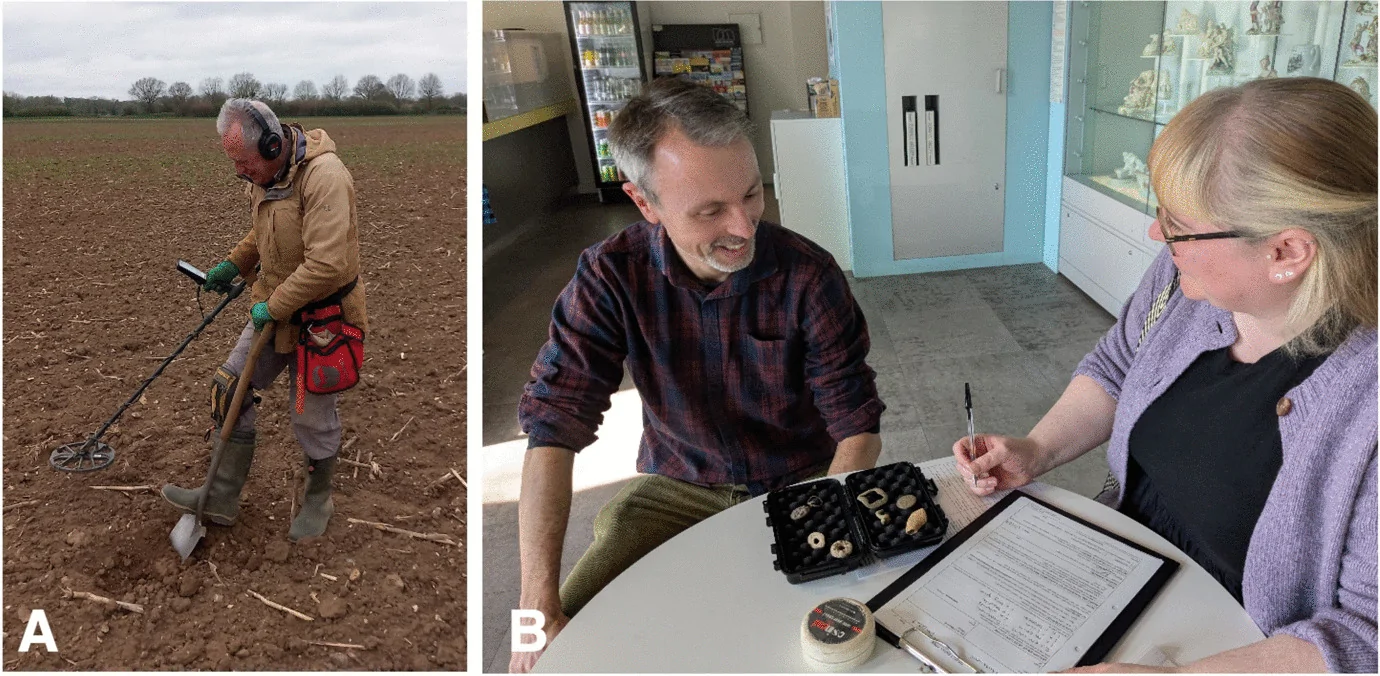
**A** AC metal detecting in a field, England, April 2026. **B** Archaeological objects of different periods recovered by a metal detectorist being recorded by a PAS Finds Liaison Officer, 2026. Photo credit: Pat Buckley (**A**), Ian Richardson (**B**)

These considerations and workflows are timely, as metal-detected finds have emerged as an increasingly important source of archaeological information over the last decade. Hobby metal detecting is legal in many European countries (Deckers et al., 2016; Dobat et al., 2020; Lewis, M., et al., 2026; Oksanen et al., 2025; Wessman et al., 2023), though certainly not all. There is now such a considerable volume of public finds records to legitimise discussion of it as cultural heritage ‘big data’ (*e.g.* Gosden et al., 2021, Oksanen & Wessman, 2025), even though it still does not usually present the kinds of challenges posed by a strict definition of this term (Bevan, 2012: 492; Kitchin & McArdle, 2016). Some sense of the large scale involved is given by the fact that of the over four million archaeological records opened by the recently launched ARIADNE Linked Open Data portal for European and global archaeology (Richards & Niccolucci, 2019; Aspöck & Richards, 2023), somewhere between one quarter and one third are metal-detected finds. These records mostly come from just three countries: the UK, the Netherlands and Denmark. These countries have well-established finds recording schemes that are capable of cooperating with hobby detectorists, ingesting their reports and turning them out in the form of publicly available research data (for Denmark see Dobat et al., 2019, for the Netherlands Kars & Heeren, 2018; Carpentier, 2022; Fig. 11). We would observe that a culture of responsible and responsive archaeological metal detecting is fostered by such cooperation, one in which the case for its value is clearly presented to all stakeholder groups including hobby detectorists (see Dobat et al., 2020; Oksanen et al., 2025).

Naturally, there is no one-size-fits-all approach, but similar schemes are also being developed in Norway, Belgium, the Czech Republic, Estonia, Finland and elsewhere (Pajdla et al., 2023; Wessman et al., 2023; Lewis, M., et al., 2026). The point we want to emphasise is that if and once other European countries develop robust schemes for recording and disseminating metal-detected finds data—something that is definitely happening right now—the finds numbers will rapidly continue to go up and up. The end result is that in many countries, archaeological scholarship is playing catch-up with this veritable flood of new finds data. What is, of course, necessary is a great deal of qualitative research to better understand the use histories, chronologies and typologies of object types that were previously not so well represented in the archaeological record. It is also important to develop open tools that can take advantage of the data’s capacity of comparatively investigating wide-ranging phenomena. Just one good example of where wider use of large quantities of metal-detected data could yield important insights at a grand scale is the Black Death and the subsequent demographic and settlement change, processes that are shared at a transnational level. Similarly, assessments of local detectorists’ scatters could be very useful for development control and for informing planning decisions (*cf*. Boldrini, 2006)—heritage management concerns that are relevant across different countries—all the more so for areas where other information is sparse. 

From a data science perspective, this research has focused on data exploration in an open-source statistical and programming environment R (R Core Team 2021) to determine whether local PAS assemblages are capable of shedding light onto possible long-term continuities in their landscape reuse. The proposed approach formalises a spatial statistical workflow that can be fine-tuned to different spatial scales, assists fieldwork by identifying sites and areas for further research, and contextualises patterns in the finds data that are relevant to the research questions posed. It quantifies, visualises and—importantly—assists in reliably communicating archaeologically relevant spatial statistical patterns that emerge from findspot distribution data. Metal detectorists will often go to places where professional archaeologists have not conducted excavations, and hence, the evidence that they produce often offers wholly new information about the past. To fully utilise the potential for this data, both for larger- and smaller-scale studies, it is clear that we need to develop new, robust and easily transferable approaches.

## Supplementary Information

Below is the link to the electronic supplementary material.

ESM 1 (TXT 12.6 KB)

## Data Availability

The public finds record data that this paper uses for analytical case studies are available from the PAS, but restrictions apply to the availability of these data. While general information about finds is released under CC-BY license by the PAS via its website (https://finds.org.uk/database), the precise coordinate locations are not publicly released in order to protect potentially vulnerable archaeology. These data, however, can be available after signing a data release agreement with the PAS (register an online user account through the link above and apply to the PAS at info@finds.org.uk providing an explanation of research use).

## Footnotes

1 PAS website: https://finds.org.uk/. The research data dump used in this study was downloaded on 29th June 2023 as part of the Medieval Ritual Landscapes project at the University of Reading and the British Museum.

2 One of the authors of this paper.
